# The dynamics of recovery and growth: how defoliation affects stored resources

**DOI:** 10.1098/rspb.2013.3355

**Published:** 2014-05-22

**Authors:** R. R. L. Atkinson, M. M. Burrell, K. E. Rose, C. P. Osborne, M. Rees

**Affiliations:** Department of Animal and Plant Sciences, University of Sheffield, Western Bank, Sheffield S10 2TN, UK

**Keywords:** life history, defoliation, relative growth rate, root storage

## Abstract

Growth rate varies widely among species and the trade-off between growth rate and storage or maintenance traits is a principal axis of variation between species. Many plant species have substantial root stores, but very little is known about how growth rate modifies responses of these stores to defoliation and other stresses. Species with different growth rates are predicted to respond in distinct ways, because of variation in the pre-defoliation allocation to storage. Here, we quantified the dynamics of stored carbohydrates in seven species with varying growth rate, following defoliation in a pot experiment. For faster growing species, there was significant reduction in carbohydrate concentration following defoliation, followed by relatively fast recovery, whereas for slower growing species, carbohydrate concentration levels remained relatively invariant across treatments. Results for total carbohydrates mirrored those for concentration, but were not as significant. Our findings were consistent with the idea that faster growing species respond more rapidly than slower growers to defoliation, through changes in carbohydrate pool concentrations. Growth rate as an indicator of life-history and ecological strategy may therefore be key to understanding post-defoliation recovery and storage strategies.

## 1. Introduction

Growth rate varies widely among species, and part of this variation is linked to variation in allocation to storage, defence and maintenance [1–4]. Previous work has shown that allocation to storage reduces maximum potential growth rates [5,6], which might seem maladaptive as rapid growth allows plants to quickly increase in size and so effectively exploit resources both above and below ground. However, in highly disturbed environments, storage is important in allowing re-growth following destruction of plant tissue, and theoretical models predict higher allocation to storage in productive, highly disturbed environments [7].

Since the allocation of resources to storage leads to a reduction in growth rate, fast-growing plant species typically have smaller stores, both in terms of absolute size and percentage of root metabolites allocated to carbohydrates [6]. This means that we might expect plant responses to defoliation to be mediated by growth rate. However, there are currently no clear theoretical predictions for understanding how stored resources should be used following defoliation, and whether this response should vary between fast- and slow-growing species.

Following defoliation, plants deploy stored resources to rebuild photosynthetic material, but how should the use of stores vary among species? Clearly, the size of the store, the construction costs of new photosynthetic material and the flexibility of the growth strategy are likely to influence the re-growth strategy. Fast-growing species typically have: (i) low leaf construction costs and (ii) more flexible growth strategies [8–10], and so we might expect them to rapidly deploy stores. This use of stores will, however, be constrained by the small amount of material stored. By contrast, slow-growing species have relatively large stores, but less flexible growth strategies and higher leaf construction costs [11,12].

These observations lead to two distinct but contrary predictions. Fast-growing species will either: (i) more rapidly use their stores as a result of their more flexible growth strategies and lower leaf construction costs than slow-growing species or (ii) have more conservative re-growth strategies as they are constrained by the small amount of material stored. Slower growing plants, with a large store of carbohydrates, may respond to defoliation by rapidly deploying their root stores to re-grow photosynthetic material or, alternatively, use of root stores may be more gradual, in accordance with the idea that slow-growing species have relatively inflexible growth strategies and higher leaf construction costs.

Here, we report the results of an experiment on seven monocarpic (once-flowering) plant species. This relatively simple life history is ideal for understanding links between growth, survival, storage and defoliation, since energy stores are only allocated to reproduction in the final terminal reproductive event. The individuals in this study were sampled from a larger experiment, where a growth–survival trade-off was established for these species following multiple, full defoliation events [13]. This trade-off may be linked to the depletion of root carbohydrate stores owing to defoliation. In order to investigate the influence of growth rate on the responses of root reserves to defoliation, we used a size-standardized measure of species growth [13,14] to classify the species as slow, medium or fast growing. To quantify how stored carbohydrate pools changed following defoliation in slow versus medium and fast growers, we completed an outdoor pot experiment. We defoliated a subset of plants either once or twice, and measured root storage (concentration and total pool size), using a non-targeted metabolomics approach for comparison with non-defoliated controls.

Based on the two contradictory predictions for how growth rate mediates the response to defoliation, we hypothesized that in the weeks following defoliation, species in each growth category would respond differently in terms of changes in (i) root carbohydrate concentrations and (ii) total root carbohydrate. We focused mainly on carbohydrate concentration, rather than total root pools, since concentration is easier to estimate accurately, although the patterns should be similar in both. We considered a reduction in carbohydrate concentration relative to controls following defoliation to be indicative of storage being used for re-growth. Previously, we found that slower growing species had higher survival following multiple defoliation events [13], and so we expect total root carbohydrates to be significantly lower for fast, compared with slow-growing defoliated plants, if stores mediate the growth–survival trade-off.

## 2. Material and methods

The experiment took place at Tapton Experimental Gardens, University of Sheffield. Seeds of *Cirsium vulgare*, *Digitalis purpurea*, *Verbascum thapsus*, *Verbascum blattaria, Carduus nutans, Arctium minor* and *Senecio jacobaea* were sown between 15 and 21 March 2007 into degradable pots 70 mm in diameter and put into a greenhouse. The pots were filled with a 9 : 1 : 1 mixture of sand: vermiculite : M3 compost. After a few weeks of growth, the plants were transferred into pots 2.2 l in volume, filled with the same sand, vermiculite and compost mixture as before, and placed outside into a randomized, eight block design, roughly balanced by species. Before the first treatment, plants were allocated to a control or treatment group. The latter consisted of one (T1) or two (T2) full defoliations (removal of all aboveground material). The first defoliation took place on 30/06/07 (dd/mm/yy). T1 plants were then left to grow until harvesting. T2 plants were defoliated a second time on 07/08/07, after full or partial re-growth of most of them had occurred (table 1). Plant size was tracked non-destructively by measuring the length of the longest leaf (table 1). Where possible three to four plants per species per treatment were harvested (*n* = 266) on the following dates: 12/05/07 (H1) (*n* = 26), 22/06/07 (H2) (*n* = 22), 7–8/07/07 (H3) (*n* = 49), 21–22/07/07 (H4) (*n* = 43), 31/08/07 (H5) (*n* = 60) and 05/03/08 (H6) (*n* = 66) (table 1). Harvests H1 and H2 comprised only control plants, H3 and H4 included control and T1 plants, and H5 and H6 encompassed control, T1 and T2 plants (table 1). Total sample size by treatment was: *C* = 150, T1 = 76 and T2 = 40.

**Table 1.** Summary of census dates for non-destructive measures of the longest leaf length, (see Rose *et al.* [13] for how these measures were used to calculate size-standardized relative growth rate values), defoliation times (vertical lines) and destructive harvests (H1–H6).

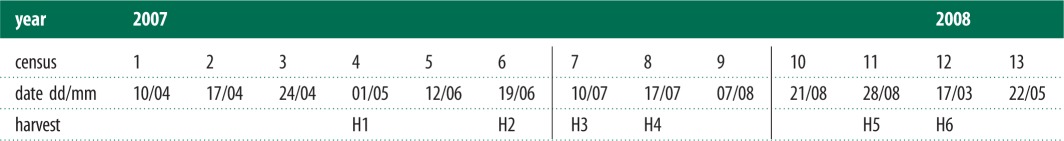

At harvest, a small cross section of taproot was removed from all plants, and flash-frozen in liquid nitrogen. These samples were stored at −80°C until extraction. The remaining roots were separated into taproot and fibrous root before taking fresh-weight measurements.

### (a) Mass spectrometry

Mass spectrometry is a method of compound detection within biological samples, by separating ions according to mass/charge (*m/z*) ratios. The output is a list of *m/z* values, each representing an individual ion, alongside the corresponding count for the ion. The relative ion count corresponds to the concentration of the ion in the biological sample relative to all other ions detected. The plant root samples were extracted using a 5 : 2 : 2 methanol : chloroform : water solution [6]. The metabolite profiles were produced over a mass range of 100–1000 Da, using an electro-spray method with a quadrupole mass spectrometer (API sciex III plus, AB Sciex UK Limited, Phoenix House Lakeside Drive, Cheshire). Samples were analysed in triplicate.

### (b) Storage compound analysis

We previously showed that sucrose and the raffinose series of carbohydrates, consisting of raffinose, stachyose and verbascose are the main carbon storage compounds in the seven species [6], and these were targeted for analysis. Peak height values were extracted from the peak centred, unbinned mass spectra data for *m/z* values corresponding to sucrose, raffinose, verbascose and stachyose. We chose the highest peak within ±0.2 Da of the monoisotopic value corresponding to the hydrogen, potassium and sodium adducts of the metabolites. A single value for each of these compounds' ions in a sample was calculated, which represented the number of ion counts for an ion in 0.1 g of taproot (‘carbohydrate concentration’ in the analyses), averaged over the three replicates. These were the data used for the individual storage compound concentration analyses. To obtain a value for the total carbohydrate concentration, we added the values for sucrose, raffinose, verbascose and stachyose, after correcting for their relative ionization energy values. Relative energy correction was possible since the basic structure of these compounds is conserved; the raffinose series is extended through addition(s) of galactose units onto a sucrose molecule. The total carbohydrates in the taproot data were calculated by scaling up the carbohydrate concentration values to the full taproot, by multiplying by the total fresh weight of the taproot. The mass spectrometer counts shown in the figures are directly proportional to the concentration of compound in the sample.

### (c) Species growth categorization

In order to correct for the size-dependence of growth rate, we fitted a nonlinear mixed effects model to the size census data using non-destructive size measurements of longest leaf length (table 1) [6,13]. In the dataset used in the present investigation, growth curves for each individual could not be estimated accurately, because destructive harvesting was limited to a small number of census dates. However, as the individuals in this experiment were sampled from a larger experiment [13], we used this larger sample of individual growth curves (*n* = 842), to calculate species-average growth rates and their standard errors [6]. These values, therefore, do not represent maximum intrinsic growth rates for each species, but the species-average growth rates for the conditions experienced by the individuals from this experiment.

### (d) Statistical analysis

All statistics were completed using the R statistical package [15]. We used ANOVA with growth category (fast, medium, slow), harvest (H1–H6) and treatment (control, one defoliation, two defoliations) and all interactions between them as fixed explanatory variables. The response variables were carbohydrate concentration, and total carbohydrates in the taproot. The total carbohydrates in the taproot data were calculated by scaling up the carbohydrate concentration values to the full taproot, by multiplying by the total fresh weight of the taproot.

All response variables were transformed using the Box-Cox transformation so the data conformed to the assumptions of ANOVA. *A priori* hypotheses were tested using planned contrasts from the fitted analysis of variance model. Block was initially added to the model, but was removed, as it was not significant in all analyses.

## 3. Results

### (a) Species-average growth rates

For the analyses, *S. jacobaea*, *C. vulgare* and *A. minor* were categorized as slow growers relative to *V. blattaria* and *C. nutans*, which were classified as medium growers, and *V. thapsus* and *D. purpurea* were classified as fast growers, based upon species-average growth rate values (table 2). This categorization of species was used in all hypothesis testing.

**Table 2.** Categorization of species with differing mean size-standardized relative growth rate, i.e. ‘fast-’, ‘medium-’ and ‘slow-'growing species. In Rose *et al*. [13], we used nonlinear mixed effects models to fit growth curves for individual plants (*n* = 842), and individual values over species were averaged to achieve a size-standardized measure of species mean growth rate at census 6, before defoliation.

species	species mean growth rate (mm/mm/day) ± the standard error around that estimate	growth category
*Verbascum thapsus*	0.022820 ± 0.0002	fast
*Digitalis purpurea*	0.020778 ± 0.0002	fast
*Carduus nutans*	0.019100 ± 0.0004	medium
*Verbascum blattaria*	0.018027 ± 0.0003	medium
*Arctium minor*	0.015869 ± 0.0006	slow
*Cirsium vulgare*	0.015883 ± 0.0006	slow
*Senecio jacobaea*	0.015804 ± 0.0003	slow

### (b) Carbohydrate concentration

**Figure 1.**
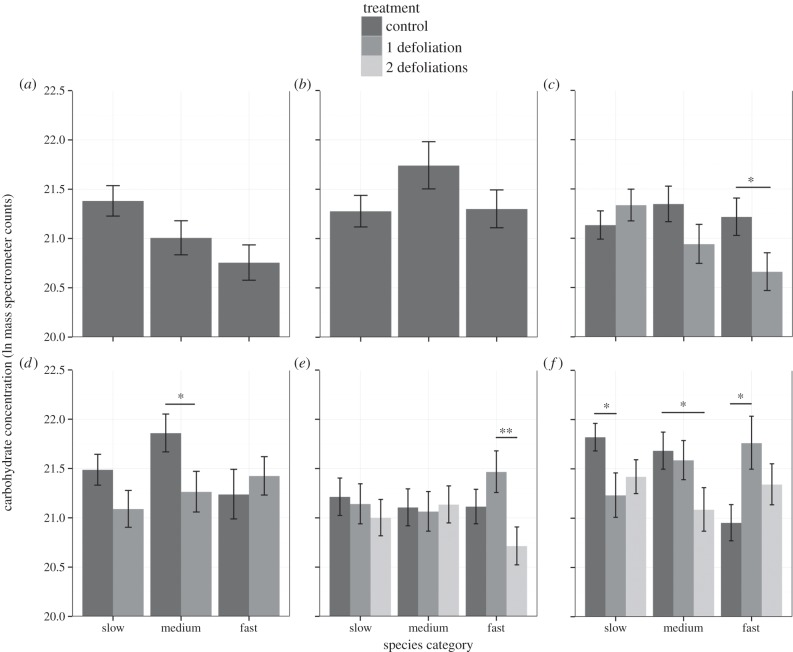
(*a*–*f*) The relationship between growth rate category and treatment (control, one defoliation and two defoliations) over harvests H1–H6. Significance values *p < 0.05, **p < 0.01, ***p < 0.001. Means and standard errors were plotted from the predicted model results. Significance information was extracted from within ANOVA *t*-tests.

Carbohydrate concentration showed a complex pattern across treatments, and between species and harvests with a significant three-way interaction between harvest, treatment and growth category (figure 1 and table 3).

**Table 3.** Analysis of variance results for root carbohydrate concentration and total root carbohydrates. The response variables were transformed using Box-Cox transformation. *F*-values are given in the body of the table, with their associated levels of statistical significance indicated by asterisks. RGR = relative growth rate.

	d.f.	carbohydrate concentration	total root carbohydrates
RGR	2	4.16*	93.83***
harvest	5	6.21***	33.94***
treatment	2	2.53	10.9***
RGR × treatment	4	1.63	0.72
RGR × harvest	10	1.07	1.71
RGR × harvest × treatment	8	2.97**	1.96
R^2^ value for model		0.17	0.62
residuals		230	202

*p < 0.05, **p < 0.01, ***p < 0.001.

Hypothesis: fast- and slow-growing plants would respond differently to defoliation treatments through changes in carbohydrate concentration after defoliation.

To test this hypothesis, in harvests H3 and H4, we compared the carbohydrate concentration between controls versus once-defoliated plants in fast-, medium- and slow-growing species (figure 1 and table 3). In harvest H3 (one week after the first defoliation), defoliated fast growers had reduced carbohydrate concentrations by 43% compared with control plants (d.f. = 230, *t* = 2.07, *p* < 0.05) but both slow- (d.f. = 230, *t* = 0.94, n.s.) and medium-growing (d.f. = 230, *t* = 1.51, n.s.) defoliated plants did not. Therefore, after defoliation faster growers more rapidly mobilized carbohydrate stores from the root than slower growing species. This supports our hypothesis that fast and slow growers would respond differently to defoliation.

In harvest H4 (two weeks after the first defoliation treatment), there were still no differences in carbohydrate concentration for control versus defoliated plants of slow-growing species (d.f. = 230, *t* = 1.62, n.s.). However, in the medium growth rate category defoliation significantly lowered carbohydrate concentrations, by 45% compared with controls, indicating the use of some root stores (d.f. = 230, *t* = 2.11, *p* < 0.05). By contrast, for fast-growing species at harvest H4, defoliation did not significantly affect carbohydrate concentration compared with controls (d.f. = 230, *t* = 0.59, n.s.). This suggests that, within two weeks of defoliation, root stores had been replenished by carbon derived from photosynthesis since defoliation. In summary, fast-growing plants had reduced carbohydrate concentrations immediately after the defoliation at harvest H3, and then medium-growing defoliated plants responded similarly at harvest H4, while slow-growing defoliated plants showed no response at either harvest.

To uncover whether the patterns in harvests H3 and H4 (after the first defoliation) were repeated in harvests H5 and H6 (after the second defoliation), we compared differences in mean carbohydrate concentrations between once-defoliated and twice-defoliated plants for fast-, medium- and slow-growing species. If twice-defoliated plants had lower carbohydrate concentrations than once-defoliated plants left to grow, then this would support the idea that carbohydrate stores had been mobilized from the root for shoot re-growth in the twice-defoliated plants.

In harvest H5 (three weeks after second defoliation), twice-defoliated plants of fast-growing species had significantly reduced mean carbohydrate concentrations by 53% in comparison to once-defoliated plants (d.f. = 230, *t* = 2.64, *p* < 0.01), but this was not the case for slow- (d.f. = 230, *t* = 0.51, n.s.) and medium-growing species (d.f. = 230, *t* = 0.26, n.s.). These results suggest that, after a second defoliation, fast-growing species mobilized some of the reserves that had accumulated after the first defoliation.

In harvest H6 (approx. six months after second defoliation), there were no differences in carbohydrate concentration between once-defoliated versus twice-defoliated plants in any growth category; for slow (d.f. = 230, *t* = 0.66, n.s.), medium (d.f. = 230, *t* = 1.68, n.s.) or fast growers (d.f. = 230, *t* = 1.24, n.s.). This indicates that, for fast-growing species, the twice-defoliated plants had recovered carbohydrate concentrations to that of the once-defoliated plants (six months after the second defoliation), repeating the pattern seen for the first defoliation.

In harvest H6, the comparison between control and defoliated plants within each growth category showed that for slow-growing species, defoliated plants had a significantly reduced mean carbohydrate concentration, for controls versus once-defoliated plants (d.f. = 230, *t* = 2.19, *p* = 0.03) and non-significantly between controls versus twice-defoliated plants (d.f. = 230, *t* = 1.8, *p* = 0.07). For medium-growing species, once-defoliated plants had similar carbohydrate concentrations to controls (d.f. = 230, *t* = 0.35, n.s.), but twice-defoliated plants had significantly reduced mean carbohydrate concentrations compared with controls (d.f. = 230, *t* = 2.05, *p* = 0.04). For fast growers, defoliated plants had increased carbohydrate concentrations relative to the controls, and this was significant for once-defoliated plants (d.f. = 230, *t* = 2.53, *p* = 0.012), but not for twice-defoliated plants (d.f. = 230, *t* = 1.41, n.s.).

### (c) Total root carbohydrates

Hypothesis: fast- and slow-growing plants respond differently to defoliation treatments through changes in total root carbohydrates.

**Figure 2.**
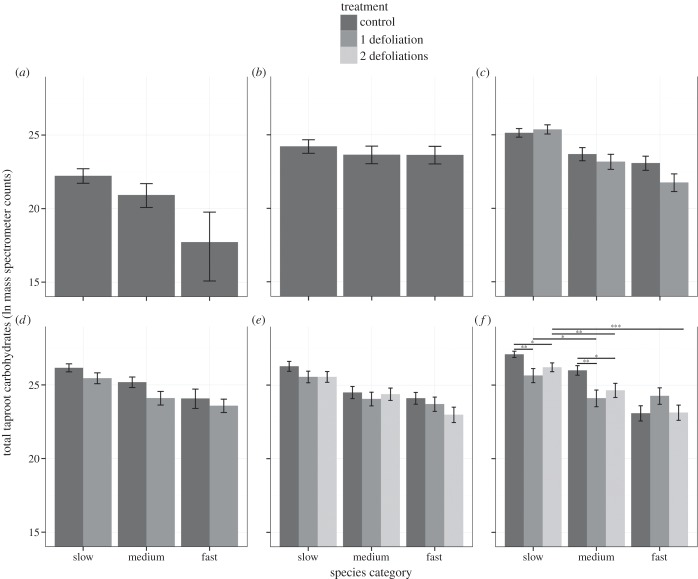
(*a*–*f*) The relationship between total root carbohydrates, growth rate category and treatment (control, one defoliation and two defoliations) over harvests H1–H6. Significance values *p < 0.05, **p < 0.01, ***p < 0.001. Means and standard errors were plotted from the predicted model results. Significance information was extracted from within ANOVA *t*-tests.

To test this prediction, the difference in total root carbohydrates between controls and once-defoliated plants was compared in all growth rate categories at harvests H3 and H4 (two and three weeks after the first defoliation, respectively (figure 2 and table 3)). At harvest H3, fast growing once-defoliated plants had reduced total root carbohydrates compared with controls, although this was not significant (d.f. = 202, *t* = 1.74, *p* = 0.084). This trend was not seen in the medium growth rate (d.f. = 202, *t* = 0.76, n.s.) nor for slow growth rate species, where once-defoliated plants had more (but not significantly so), total carbohydrates compared with controls (d.f. = 202, *t* = 0.55, n.s.). At harvest H4, once-defoliated plants of medium-growing species had reduced total carbohydrates (d.f. = 202, *t* = 1.87, *p* = 0.063). This mirrors our findings for carbohydrate concentration, where once-defoliated plants of medium-growing species had a significantly reduced carbohydrate concentration compared with controls (figure 1). In harvest H4, the difference between the total root carbohydrates of controls and once-defoliated plants was not significant for species in the slow- and fast-growth rate categories.

To test whether the second defoliation resulted in an additional reduction in carbohydrate stores, we compared once-defoliated and twice-defoliated plants within each growth category at harvests H5 and H6. There was no difference in total root carbohydrates between once-defoliated and twice-defoliated plants for any growth category at either harvest.

In general, results for total root carbohydrate mirrored results found at the root carbohydrate level, as the effects were in the same direction, but the comparisons were less statistically significant.

Prediction: in the final harvest (harvest H6), we expected that (a) for each defoliation treatment, total root carbohydrates would be lower for fast-growing compared with slow-growing species and (b) in each growth category defoliated plants would have lower total carbohydrates than controls. Higher mortality for fast growers may therefore be associated with lower carbohydrate levels

To test prediction (a), we first compared the total root carbohydrate content of once-defoliated slow-growing species versus once-defoliated medium and fast growers (table 3 and figure 2). The twice-defoliated plants were then compared to test whether slower growing species had a higher total of taproot carbohydrates, as predicted. The medium growing, once-defoliated plants had significantly less, by 79%, total root carbohydrates than once-defoliated slow growers (d.f. = 202, *t* = 2.09, *p* < 0.05), and fast growers had 75% less than slow growers (d.f. = 202, *t* = 1.89, *p* = 0.06). The same comparison for twice-defoliated plants showed that medium-growing species had significantly less total root carbohydrates, by 79% than slow-growing species (d.f. = 202, *t* = 2.86, *p* < 0.01) and fast growers had 95% less than slow-growing species (d.f. = 202, *t* = 5.50, *p* < 0.001).

To test prediction (b), we compared total root carbohydrate content of control plants *versus* once-defoliated and twice-defoliated plants within each growth category. Slow- and medium-growing once-defoliated plants had reduced stores compared with respective controls, for slow growers, once-defoliated plants had 76% less (d.f. = 202, *t* = 2.90, *p* < 0.01), and for medium growers once-defoliated plants had 85% less (d.f. = 202, *t* = 3.02, *p* < 0.01). However, this comparison for fast growers showed no significant change in total stores (d.f. = 202, *t* = 1.54, *p* = n.s.). Slow- and medium-growing twice-defoliated plants also had reduced stores compared with controls; slow growers had 59% less (d.f. = 202, *t* = 2.44, *p* < 0.05) and medium growers had 74% less (d.f. = 202, *t* = 2.34, *p* < 0.05). Again, this comparison for fast growers was not significant (d.f. = 202, *t* = 0.062, n.s.).

## 4. Discussion

The changes in carbohydrate storage over harvests and between treatments were complex, but underpinned by consistent patterns within growth category groupings. We found that the concentration of root carbohydrates in the slow growth category did not alter significantly immediately following defoliation (figure 1 and table 3). By contrast, the carbohydrate concentration of species in the high growth rate category declined significantly shortly after defoliation (one week), but had recovered rapidly by two weeks after defoliation. Species in the medium growth category showed an intermediate response, with a dip in storage carbohydrate concentration two weeks after defoliation. Overall, these patterns are consistent with the idea that faster growing species have a more rapid response to defoliation than slower growing species, and our prediction that species in different growth categories would exhibit distinct post-defoliation recovery strategies.

For slow-growing species, total carbohydrate concentrations did not alter substantially within the first week following a defoliation event (control versus one defoliation in harvest H3 and one defoliation versus two defoliations in harvest H5). By contrast, species in the fast growth category had significantly reduced root carbohydrate concentrations a week following defoliation, and this trend was also apparent for medium growers. Strikingly, in subsequent harvests, fast-growing species had increased carbohydrate concentrations after defoliation relative to controls, which was not observed in other growth rate categories. We have demonstrated that re-growth strategy and the dependence on root carbohydrate pools following shoot loss may differ based on whether species are relatively fast or slow growing, and, in turn, life-history strategy. In general, slow-growing species exhibit more conservative survival strategies, for instance, through the development of organs with high tissue densities and longer turnover times [16]. A high initial investment to root storage, as opposed to shoot storage, maintains reserves in a comparatively safe below-ground compartment that excludes most herbivores. We found that this conservative approach is maintained after defoliation. The results for species in the high growth category were consistent with the idea that faster growing species generally have riskier initial allocation strategies, combined with a more prominent allocation change in response to defoliation. This meant that storage was both depleted faster after defoliation, and recharged more rapidly to higher levels in fast- than slow-growing species.

The final March harvest (harvest H6) was completed just before spring re-growth, several weeks before bolting and subsequent flowering of most individuals. A disparity in carbohydrate storage over treatment groups and growth categories at this time of year may explain why we found previously that faster growing species had higher mortality and lower flowering probabilities than slower growers [13]. We showed that the slower growing species had greater total root carbohydrate pools than the faster growing species in a comparison within each of the defoliation treatments. However, the picture is not simple at the final harvest, as *within* the fast growth category, there were no detectable differences in total carbohydrate pool sizes between defoliated plants and controls. Therefore, it seems the reduced survival in the defoliated, fast-growing species [13], cannot be explained solely by reduced total carbohydrate stores. One plausible explanation is that, in large, fast-growing species, a significant proportion of whole plant nutrient content is likely to be present in above-ground material. Our defoliation treatments removed all of the above-ground material, leading to greater resource loss in fast compared with slower growing species. This greater loss of resources in above-ground material may explain the observation in the companion paper by Rose *et al*. [13] that, following defoliation, faster growing species were subjected to higher mortality and lower flowering rates, despite having comparable total root stores.

In summary, our results suggest that the faster growing species have more flexible allocation responses to defoliation and that growth strategy underpins post-defoliation and recovery strategies. Since our results are based upon measures that allow us only to infer allocation changes, a dynamic whole-plant approach is now necessary to distinguish the effects of nutrient availability and re-allocation from different plant organs. Our study focused on monocarpic perennials, since these species have a life history that considerably simplifies the analysis and interpretation of growth and storage patterns after defoliation. In order to assess the generality of these results, further experiments with a broader range of plant life histories are required.

## Data accessibility

Data are available from the Dryad Digital Repository: http://doi.org/10.5061/dryad.c8qn1.
